# Integrating BRAF/MEK inhibitors into combination therapy for melanoma

**DOI:** 10.1038/sj.bjc.6604891

**Published:** 2009-01-20

**Authors:** K S M Smalley, K T Flaherty

**Affiliations:** 1 Molecular Oncology Department, The Moffitt Cancer Center and Research Institute, 12902 Magnolia Drive, Tampa, FL 33612, USA; 2 Department of Hematology-Oncology and The Abramson Cancer Center, University of Pennsylvania School of Medicine, Philadelphia, PA 19104, USA

**Keywords:** melanoma, targeted therapy, BRAF, PI3K, mTOR, PTEN, chemotherapy

## Abstract

The discovery of BRAF mutations in melanoma has not yet translated into clinical success, suggesting that BRAF/MEK inhibitors will need to be combined with other agents. In the current review, we discuss other pathways likely to be important for melanoma progression and suggest possible drug combinations for future clinical testing.

The discovery of activating oncogenic mutations in *BRAF* in over 60% of melanomas has raised expectations that melanoma may be amenable to targeted therapy. The most prevalent mutation in *BRAF* is at the V600E position, resulting in the substitution of valine to glutamate and destabilisation of the inactive kinase conformation switching the equilibrium towards the active form (Gray-Schopfer *et al*, 2007). Most of the transforming activity of the *BRAF* V600E is thought to result through the activation of the mitogen-activated protein kinase (MAPK) pathway (Gray-Schopfer *et al*, 2007). Regardless of *BRAF* V600E mutational status, virtually all melanomas have activity in the MAPK pathway (Satyamoorthy *et al*, 2003), and this contributes to the oncogenic phenotype of melanoma through its effects on cell proliferation, invasion and survival (Gray-Schopfer *et al*, 2007). In experimental systems, the role of *BRAF* in melanoma seems convincing. *In vitro* studies have shown that *BRAF* V600E is an oncogene in immortalised mouse melanocytes (Gray-Schopfer *et al*, 2007), and the selective downregulation of *BRAF* V600E using RNAi causes cell death and reversal of the melanoma phenotype (Hingorani *et al*, 2003). However, pharmacological inhibition of BRAF and MEK have not produced such dramatic effects *in vivo* (Sharma *et al*, 2005; Haass *et al*, 2008). This is partly due to inherent flaws in the agents tested to date, and also a likely indication of the greater vulnerability of melanoma cells *in vitro* where most of the cells are rapidly cycling under sub-confluent culture conditions in the continuous presence of serum.

## Preclinical studies on braf/mek inhibitors

The first putative BRAF inhibitor to be developed was sorafenib (Nexavar®, BAY 43-9006). A number of studies have now shown that sorafenib induces melanoma cell apoptosis *in vitro* and reduces the growth of human melanoma xenografts in mice (Sharma *et al*, 2005). Although it was shown that phospho-MEK was blocked at the concentrations of sorafenib used, only relatively minor levels of apoptosis were observed *in vivo*, suggestive of alternative mechanisms of action. It was later shown that sorafenib treatment reduced the vascularisation of melanoma xenografts (Sharma *et al*, 2005), and that the compound was a relatively potent VEGF receptor inhibitor.

A number of more specific BRAF inhibitors have now been developed, some of which have relative selectivity for the *BRAF* V600E mutation compared with wild-type *BRAF*, such as SB590885 (GlaxoSmithKline, Collegeville, PA, USA) (King *et al*, 2006) and PLX-4032/PLX-4720 (Plexxikon, Berkley, CA, USA) (Tsai *et al*, 2008). As selective BRAF targeted compounds have relatively few off-target effects, it is now possible to assess the effects of specific pharmacological inhibition of BRAF in melanoma. An extensive characterisation of SB590885 has shown that the compound is highly selective for cell lines with *BRAF* mutations, accompanied by a profound inhibition of cell growth associated with the induction of G1-phase cell cycle arrest (King *et al*, 2006). Interestingly, SB590885 activity against human melanoma xenografts in mice is fairly weak, and there is merely a delay in the onset of tumor growth. The interpretation of these results is somewhat hindered by the poor pharmacological properties of this agent. Similar G1-phase growth arrest results have been observed with allosteric MEK inhibitors, such as U0126, PD0325901 and AZD6244, suggesting that inhibition of the MAPK pathway in melanoma is largely cytostatic (Smalley *et al*, 2006; Solit *et al*, 2006; Haass *et al*, 2008). Activating *BRAF* V600E mutations are also known to occur in subsets of thyroid and colon carcinomas (Davies *et al*, 2002), and similar to melanoma, the presence of the mutation also predicts for sensitivity to MEK inhibition (Solit *et al*, 2006; Leboeuf *et al*, 2008). There is evidence that PLX-4032 and its tool-compound counterpart PLX-4720 induces some limited apoptosis in melanoma cell lines with the V600E mutation but not in those that are *BRAF* wild type (Tsai *et al*, 2008). Given the pivotal role of *BRAF* in melanoma progression, it is somewhat surprising that these pharmacological inhibitors do not generally induce much apoptosis. Recent studies have shown that blocking the MAPK pathway did not affect the levels of Bcl-2, Bcl-XL or Mcl-1 expression (Verhaegen *et al*, 2006). Knockdown of the BH3-family protein Mcl-1 using shRNA sensitised the melanoma cells to U0126-induced apoptosis, showing that overexpression of these key anti-apoptotic proteins in melanoma is a critical barrier to an effective BRAF/MEK inhibitor therapy. It is therefore suggested that melanoma cells recruit additional survival mechanisms that are MAPK pathway-independent.

## Why combination therapies are needed in melanoma

**Figure 1:**
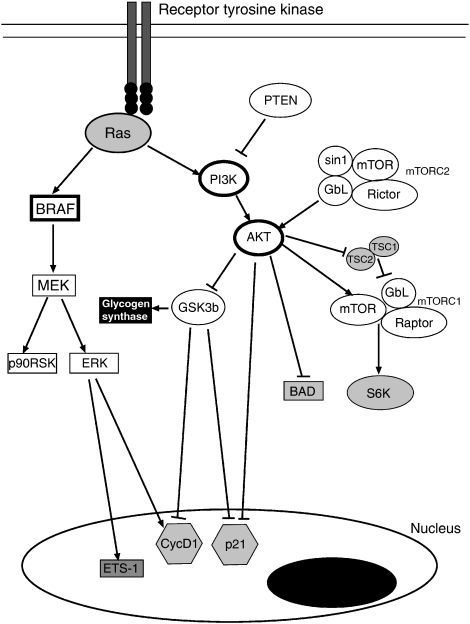
Sample signalling scheme showing pathways known to be active in melanoma. Preclinical studies support the combined targeting of either BRAF/MAPK and PI3K/AKT pathway or the BRAF/MAPK and mTOR signalling pathways in melanoma. It is likely that the combined inhibition of BRAF/PI3K pathways will have synergistic effects at the level of growth inhibition, via effects upon cyclin D1 expression, as well as through increased expression of the cyclin dependent kinase inhibitor p21^waf-1/cip-1^. Inhibition of the AKT/mTOR pathway is likely to affect cell survival via modulation of BAD/Bcl-2, as well as affecting protein translation at the level of S-6-kinase (S6K).

It is likely that as the melanomas progress, there is functional redundancy between the numerous signalling pathways (Figure 1). Earlier studies from our laboratory, using cell lines derived from metastatic lesions, confirm this hypothesis and demonstrate that cells are resistant to both MEK and PI3K inhibitors when grown in 3D culture (Smalley *et al*, 2006). It was further shown that targeting either the PI3K or MEK pathway alone led to cytostasis and was associated with a reversible G1-phase cell cycle arrest (Smalley *et al*, 2006; Haass *et al*, 2008). In other studies, there was a lack of good correlation between the inhibition of either the PI3K or MEK signalling pathway and the inhibition of cell growth, showing a functional redundancy between the various growth-promoting signalling pathways. These findings also translated into *in vivo* studies, where the MEK inhibitor AZD6244 led to the stabilisation of established human melanoma xenografts, but not tumor regression (Haass *et al*, 2008). Furthermore, this suggests that the effects of MEK inhibition in this setting were largely cytostatic. This lack of good cytotoxic activity after either MEK or PI3K inhibition alone suggests that multiple signalling pathways need to be targeted simultaneously to induce melanoma regression.

It is currently unclear how well the preclinical findings on the role of *BRAF* in melanoma cell lines match with the clinical observations on the role of *BRAF* in melanoma pathogenesis. A number of reports have suggested that the levels of phospho-ERK staining are often variable in patient tumors and do not correlate with the *BRAF/NRAS* mutational status (Houben *et al*, 2008). Even in melanomas that are the most sensitive, prolonged BRAF/MEK inhibitor treatment may lead to the rapid acquisition of drug resistance. Earlier studies have shown that prolonged treatment (>6 months) with the MEK inhibitor CI-1040 leads to a 100-fold loss of potency in apoptosis and colony formation assays associated with increased expression of activated KRAS (Wang *et al*, 2005). Some of the first reports have now appeared indicating that melanoma cells can also become resistant to BRAF inhibitors after continuous drug exposure. Treatment of melanoma cells with the BRAF inhibitor AZ628 led to the development of clones that maintained high phospho-ERK levels and continued to proliferate in the presence of drug (Montagut *et al*, 2008). In this instance, resistance was associated with a switching from BRAF to CRAF signalling, an effect that could be reversed by treatment with the heat shock protein (HSP)-90 inhibitor geldanamycin (Montagut *et al*, 2008). There is also recent evidence from our group demonstrating that some melanomas with *BRAF* V600E mutations may be intrinsically resistant to inhibitors of BRAF as a result of cyclin D1 amplification (Smalley *et al*, 2008).

The key question facing the field is what combination of signal transduction inhibitors are needed to achieve the maximal cytotoxic effect, and whether the combination of inhibitors is determined by the underlying genetics of the tumor?

## Suitable combination targets: the PI3K pathway?

Mitogen-activated protein kinase is not the only pathway to be constitutively active in melanoma (Figure 1). Another key pathway involved in cell survival, growth and apoptosis suppression is the PI3K/AKT pathway. Melanomas generally lack *PI3K* and *AKT* mutations, but PTEN is lost in 30% of cell lines and ∼10% of clinical melanoma specimens. Recent studies have shown that AKT is able to transform melanocytes under hypoxic conditions (Bedogni *et al*, 2005), and there is evidence of cooperation between *BRAF* V600E and AKT in melanoma development (Cheung *et al*, 2008). Of the three AKT family members (AKT1–3), about 50% of melanoma cells have constitutive activity in AKT3 (Stahl *et al*, 2004). Inhibition of AKT activity in melanoma, using either PI3K inhibitors or selective RNAi to AKT3, or both, reduces growth and induces some degree of apoptosis (Stahl *et al*, 2004). There is preclinical evidence to suggest that there is a synergistic anti-melanoma activity when MEK and PI3K/AKT are inhibited concomitantly. Thus, the topical application of U0126 and LY294002 led to the regression of xenografts in a TPRas-induced melanoma model (Bedogni *et al*, 2004) associated with increased apoptosis and a reduced angiogenic response. Other studies have shown that PI3K and MEK inhibitors synergise to reduce growth and survival of melanoma cell lines grown under 3D organotypic cell culture conditions (Smalley *et al*, 2006; Meier *et al*, 2007). There is also evidence that siRNA knockdown of AKT3 and *BRAF* V600E leads to the enhanced inhibition of melanoma xenograft growth in nude mice (Cheung *et al*, 2008; Tran *et al*, 2008).

## Suitable combination targets: mtor signalling?

The mammalian target of rapamycin (mTOR) is a kinase that occupies a pivotal position in growth factor receptor and nutrient availability signalling, and has important downstream effects on cell growth and survival. It is also a part of the AKT signalling pathway and regulates the activity of this kinase in complex ways. Mammalian target of rapamycin can either positively or negatively regulate AKT signalling, and this is dependent on the composition of the mTOR signalling complex. Thus, mTOR forms at least two protein signalling complexes called mTORC1 and mTORC2. mTORC1 consists of mTOR, as well as the regulatory proteins raptor and mLST8. Activation of the mTORC1 signalling complex leads to the phosphorylation and activation of p70 S6kinase (S6K) and the eukaryotic initiation factor 4E-binding protein (4EBP1) (Reiling and Sabatini, 2006). In contrast, the mTORC2 complex also consists of mTOR and mLST8, and additionally includes the regulatory proteins rictor and mSin-1. The differences between the two mTOR complexes are highly relevant, as an active mTORC1 complex suppresses AKT signalling, whereas mTORC2 stimulates AKT signalling through a phosphorylation event. A number of small molecule inhibitors are available that target mTORC1 but not mTORC2 signalling. The best characterised of these is rapamycin, a drug which is currently FDA-approved for use as an immunosuppressive agent after organ transplantation. There is evidence from the literature that mTOR signalling is active in melanoma. Studies have shown the presence of constitutive phospho-S6K in a high proportion of metastatic melanoma samples (Karbowniczek *et al*, 2008). Further work has shown that rapamycin has growth inhibitory effects across a panel of human melanoma cell lines (Molhoek *et al*, 2005), and there is evidence of synergistic pro-apoptotic activity between sorafenib/MEK inhibitors and rapamycin in preclinical models of melanoma (Molhoek *et al*, 2005; Meier *et al*, 2007; Lasithiotakis *et al*, 2008). Mechanistically, it seems that combined MEK/mTOR inhibition reduces the expressions of Mcl-1 and Bcl-2, two proteins that are known to suppress apoptosis induction in melanoma cells (Lasithiotakis *et al*, 2008).

## Suitable combination targets: chemotherapy

Although most attention preclinically is focused on the combined inhibition of signal transduction pathways, most clinical interest is focused on the combination of targeted therapy agents with chemotherapy. Already, there are early suggestions that sorafenib may enhance the activity of carboplatin/paclitaxel and dacarbazine (McDermott *et al*, 2008). From a biochemical standpoint, very little is known about how MAPK signalling may modulate responses to chemotherapy, although it is known that constitutive MAPK activity, arising from mutations in HRAS, mediates cisplatin resistance through an increased DNA repair activity (Yen *et al*, 1997). There is evidence for the role of MEK in the upregulation of the DNA repair genes XRCC1 and ERCC1 after DNA damage and that this may be mediated by the transcription factor GATA-1 (Andrieux *et al*, 2007).

Rather more is known about the interaction between MEK inhibitors and the microtubule-stabilising taxanes. Treatment of cancer cells with paclitaxel is known to activate the MAPK pathway, and inhibition of MEK in combination with paclitaxel leads to additive effects on cell growth inhibition and apoptosis induction. It has also been shown that in addition to its effect on the G1/S-phase cell cycle transition, MAPK activity is also critical during meiosis and mitosis. Activated MAPK components (including BRAF) localise to the asters and kinetochores during mitosis, as well as to the actin-microtubule cytoskeleton. Earlier studies have shown that the orally available MEK inhibitor CI-1040 enhances the efficacy of paclitaxel in lung cancer xenograft models (McDaid *et al*, 2005), and that the MEK inhibitor AZD6244 enhances the pro-apoptotic effects of docetaxel in human melanoma cells (Haass *et al*, 2008). There is some suggestion that this combination may be effective because MEK activity is required for successful execution of mitosis, and the co-administration of an MEK inhibitor and paclitaxel enhances the level of mitotic catastrophe. These combinations are now undergoing evaluation for a number of solid tumors in phase I clinical trials, and a randomised phase II trial comparing AZD6244 combined with docetaxel, with docetaxel alone is planned.

## Clinical studies on braf/mek inhibitors

The current expectation is that BRAF inhibitors will be used in combination with either other targeted therapy agents or established chemotherapy regimens. However, it is necessary to rigorously evaluate each of the novel BRAF and MEK inhibitors as single agents so that we understand their distinct pharmacological properties and their respective abilities to hit their target and perturb proliferation or cell viability. Only with this knowledge can we make an informed selection of agents for further development.

Sorafenib is the most thoroughly investigated signal transduction inhibitor in clinical trials of melanoma patients. As a single agent, it was associated with only one objective response among 61 patients (Eisen *et al*, 2006). A small subset of patients with previously progressive metastatic disease maintained stable disease for more than 6 months, but an insufficient number to support the inclusion of sorafenib into a randomised phase III trial as a single agent. Analysis of patient samples pre- and posttreatment revealed that sorafenib incompletely inhibited ERK phosphorylation. This observation may be significant as preclinical studies show that complete pathway inhibition is needed to induce cell cycle arrest (Solit *et al*, 2006; Haass *et al*, 2008). In the single-agent phase II trials with sorafenib, the common toxicities were hypertension and hand-foot syndrome, consistent with the toxicities described with inhibitors of VEGF signalling. On the basis of these observations, the inhibitory activity of sorafenib against VEGF receptors (VEGFR2, VEGFR3 and VEGFR1) was explored with dynamic contrast-enhanced MRI and the results supported such an effect.

The combination of sorafenib with either carboplatin/paclitaxel or temozolomide has suggested some benefits. Consistent with the hypothesis that sorafenib is a weak inhibitor of BRAF in humans, an association between *BRAF* mutation and benefit from sorafenib/chemotherapy regimens has not been observed (Flaherty *et al*, 2008). A small, randomised phase II trial suggested benefit when sorafenib was added to dacarbazine compared with dacarbazine alone (McDermott *et al*, 2008). A phase III trial comparing sorafenib combined with chemotherapy with chemotherapy alone in patients with melanoma has completed accrual and results are expected in late 2009. The mechanism by which sorafenib appears to enhance the cytotoxicity of chemotherapy remains in question, and is complicated by the broad-spectrum anti-kinase activity of sorafenib. Although there is evidence that MEK inhibition may sensitise cells to certain chemotherapies, it is not clear whether this mechanism is shared by sorafenib. A number of other studies have shown that inhibitors of VEGF signalling, such as bevacizumab, synergise with chemotherapy drugs, suggesting that the anti-VEGF activity of sorafenib may underlie its interaction with chemotherapy (Folkins *et al*, 2007). The broad-spectrum effects of sorafenib also leave open the possibility that anti-tumor immunity may be impaired after treatment (Hipp *et al*, 2008). As partial MAP kinase pathway inhibition, angiogenesis inhibition and suppression of anti-tumor response might combine to be effective in some patients, but counter-productive in others, significant additional work remains to be done to elucidate which patients may benefit from a sorafenib/chemotherapy approach. Some clinical studies have suggested that the presence of BRAF mutations confers a lower likelihood of response or disease control with multi-agent chemotherapy regimens (Kumar *et al*, 2003) . This is clearly an area deserving further investigation, as it is unclear whether inhibition of BRAF would reverse this resistance, or whether that property is mediated through other pathways.

The MEK inhibitors—PD0325901 and AZD6244—have also progressed through phase I, and in the case of AZD6244 phase II clinical trials. PD0325901 has been evaluated in a phase I trial, in which most of the patients had melanoma. Of these patients, some experienced an objective response (2 out of 27) and 5 additional patients showed some disease stabilisation. Dose-limiting diarrhoea and rash precluded further dose escalation and possibly preclude the delivery of a dose required to adequately suppress the MAPK pathway. Phase II trials of this drug were suspended because of the occurrence of retinal vein thrombosis in several patients. AZD6244 was evaluated in a phase I trial among patients with advanced solid tumors (Adjei *et al*, 2008). Of the melanoma patients treated, none had an objective response; however, four patients maintained disease stabilisation for more than 6 months, suggesting a clinical benefit. A randomised phase II trial was completed among 200 patients with melanoma. Patients were randomised in a 1 : 1 manner to AZD6244 or temozolomide (Dummer *et al*, 2008). Five objective responses were observed among 42 patients with *BRAF* V600E mutations (12% objective response rate), indicating that a subset of *BRAF* mutant melanomas may be sensitive to this agent. The trial was designed to detect an improvement in progression-free survival compared with single-agent temozolomide. As this activity was not seen, AZD6244 was deemed insufficient to warrant further single-agent clinical trials in melanoma.

As the preclinical data support the selective activity of RAF and MEK inhibitors in *BRAF* mutant melanoma, it is logical to accrue patients to phase II trials with these agents. This requires the elaboration of real-time mutation screening for inclusion into clinical trials, a hurdle not previously surmounted in earlier targeted therapy trials in cancer. The identification of concomitant genetic alterations or other markers of aberrant signal transduction in the same tumor samples used for *BRAF* mutation testing will set the foundation for exploration of markers of response or resistance. If a responsive subset of *BRAF*-mutated melanomas can be identified, it may be possible to rapidly develop RAF and MEK inhibitors as single agents for this population, whereas combination strategies are explored for the remaining.

Although the preclinical data are compelling in support of combining BRAF inhibition with a PI3K/AKT or mTOR inhibitor, the availability of agents targeting the mediators for phase I combination trials is severely limited. Potent and selective PI3K or AKT inhibitors are currently used in phase I clinical trials. As they emerge from phase I trials, there is the possibility of evaluating rational combinations with selective RAF or MEK inhibitors. Only rapamycin-analog mTOR inhibitors are readily available. It is not clear that these agents adequately block the upstream activation of this pathway, as they only block TORC1 complex signalling and permit enhanced activation of AKT by TORC2. A phase II trial was conducted with single-agent temsirolimus in 33 patients with metastatic melanoma unselected for any molecular or genetic features (Margolin *et al*, 2005). One partial response was observed and median progression-free survival was just over 2 months, consistent with an inactive therapy. However, there was little reason to hope that this therapy would have a stand-alone role. Rapamycin analogs are sufficiently tolerable to be evaluated in combination with other agents, and are presently the only agents one can feasibly combine with emerging BRAF or MEK inhibitors.

## Conclusion

Although MAPK activation is a key step in the oncogenic transformation of melanocytes into melanoma, it appears that only a small minority of patients will benefit from the single-agent BRAF/MEK inhibitor treatment. The redundancy within the multiple signalling pathways activated in melanoma, along with the likelihood of drug resistance, suggests that combination therapy strategies will be required for effective disease management. It is further likely that the same molecular pathways involved in melanoma progression will also play important roles in both the angiogenic and immune response of melanoma, so that the targeting of these molecular pathways may have other beneficial effects beyond growth inhibition. As melanomas are more genetically heterogeneous than first thought, it is likely that ‘personalised’ combination therapies will need to be developed, whereby patient therapy is dictated by the constellation of mutations found within their tumors. The idea of personalised cancer therapy has already been demonstrated by the use of epidermal growth factor receptor inhibitors in lung cancer, where only ∼10% of patients harbour epidermal growth factor mutations and most fit a particular profile (adenocarcinoma, never-smoker, East-Asian ethnic origin and female). Similarly, in melanoma, there are early reports of success when selected groups of patients harbouring activating mutations in the receptor tyrosine kinase *c-KIT* have been treated with imatinib (Hodi *et al*, 2008). Clearly, there is much still to do, but we believe that targeting the correct combinations of signalling pathways in carefully selected groups of patients could give the therapeutic breakthrough that has been long overdue in melanoma.
